# Mitochondria-Targeted Self-Assembly of Peptide-Based Nanomaterials

**DOI:** 10.3389/fbioe.2021.782234

**Published:** 2021-11-26

**Authors:** Zhen Luo, Yujuan Gao, Zhongyu Duan, Yu Yi, Hao Wang

**Affiliations:** ^1^ School of Chemical Engineering and Technology, Hebei University of Technology, Tianjin, China; ^2^ CAS Center for Excellence in Nanoscience, CAS Key Laboratory for Biomedical Effects of Nanomaterials and Nanosafety, National Center for Nanoscience and Technology (NCNST), Beijing, China; ^3^ Center of Materials Science and Optoelectronics Engineering, University of Chinese Academy of Sciences, Beijing, China

**Keywords:** mitochondrion, self-assembly, peptide, enzyme, nanomaterials, cancer therapy

## Abstract

Mitochondria are well known to serve as the powerhouse for cells and also the initiator for some vital signaling pathways. A variety of diseases are discovered to be associated with the abnormalities of mitochondria, including cancers. Thus, targeting mitochondria and their metabolisms are recognized to be promising for cancer therapy. In recent years, great efforts have been devoted to developing mitochondria-targeted pharmaceuticals, including small molecular drugs, peptides, proteins, and genes, with several molecular drugs and peptides enrolled in clinical trials. Along with the advances of nanotechnology, self-assembled peptide-nanomaterials that integrate the biomarker-targeting, stimuli-response, self-assembly, and therapeutic effect, have been attracted increasing interest in the fields of biotechnology and nanomedicine. Particularly, *in situ* mitochondria-targeted self-assembling peptides that can assemble on the surface or inside mitochondria have opened another dimension for the mitochondria-targeted cancer therapy. Here, we highlight the recent progress of mitochondria-targeted peptide-nanomaterials, especially those *in situ* self-assembly systems in mitochondria, and their applications in cancer treatments.

## Introduction

Mitochondria, the dynamic sub-organelles in mammalian cells, are well known to be involved in the generation of adenosine triphosphate (ATP) (Roger et al., 2017). They are composed of mitochondrial membranes that include a porous outer membrane and an inner membrane with a space, and a mitochondrial matrix inside (Frey and Mannella, 2000). With their own genome and fission signatures (Gray et al., 1999; Jiao et al., 2021; Kleele et al., 2021), mitochondria also participate in other essential physiological functions in the body, such as macromolecule biosynthesis (Spinelli and Haigis, 2018) and cell proliferation (Diebold et al., 2019), differentiation (Seo et al., 2018), apoptosis (Bock and Tait, 2020), information transmission (Chandel, 2015), etc. Dysfunctions of mitochondria are discovered to be associated with a series of diseases that threaten human health (Nunnari and Suomalainen, 2012), including neurodegeneration (Devine and Kittler, 2018), cardiovascular disease (Siasos et al., 2018), and cancer (Ward and Thompson, 2012). Increasing evidence has revealed the relevance between the energetic production, metabolic biosynthesis, and singling pathways of mitochondria with the carcinogenesis (Weinberg and Chandel, 2015). Therefore, the mitochondrion has been recognized as a promising target to improve cancer therapeutics (Fulda et al., 2010; Vasan et al., 2020; Gao et al., 2021).

Currently, mitochondria can be intervened by either using the mitochondria-targeted reagents or modulating the specific targets, gene transcriptions, and kinase activities within or outside mitochondria (Smith et al., 2012). Particularly, with the discovery of several types of targeted compounds, the mitochondria-targeted approach attracts increasing attentions (Zinovkin and Zamyatnin, 2019). For instance, lipophilic cations, such as triphenylphosphonium (TPP) and dequalinium, are first discovered to target mitochondria (Murphy and Smith, 2007). TPP contains a positively charged phosphorus atom delocalized over three hydrophobic benzene rings (Liberman et al., 1969). The unique structure allows TPP to target the mitochondrial membrane due to the negative membrane potential and the favored activation energy when crossing phospholipid bilayers, resulting in a thousand-fold enhancement in the mitochondrial accumulation (Murphy and Hartley, 2018). Till now, two TPP-based small molecular antioxidants, MitoQ and SkQ1 (Kelso et al., 2001; Skulachev, 2007), have been enrolled in clinical trials for treatments of Parkinson disease, chronic kidney disease, and hepatitis C, as well as the dry-eye syndrome, respectively (Battogtokh et al., 2018; Kang, 2018; Jeena MT. et al., 2020). The second category is the mitochondria-targeted peptides, mainly including Szeto-Schiller (SS) peptides and mitochondria-penetrating peptides (MMPs) (Horton et al., 2008; Li and Huang, 2020). These peptides usually consist of hydrophobic and positively charged amino acids, which are assumed to target mitochondria driven by the negative membrane potential and the interaction with phospholipids on mitochondrial inner membranes (Zhao et al., 2004; Birk et al., 2013; Jean et al., 2016). One formulation of SS peptide (MTP-131) has been enrolled in clinical trials for treatments of heart attack and skeletal muscle mitochondrial dysfunction in the elderly. Besides, the mitochondrial precursor protein is a natural mitochondria-targeted species, which enters mitochondria *via* the mitochondrial protein import machinery (Neupert and Herrmann, 2007). These proteins contain a cleavable N-terminal targeted sequence, which is cleaved by mitochondrial peptidases once entering mitochondria (Wasilewski et al., 2017). In recent years, self-assembled peptide-nanomaterials are emerging as a new type of mitochondria-targeted category, due to their designable feature to combine the targeting, biological responsive, self-assembling, and therapeutic properties (Qi et al., 2018).

In this review, we focus on the mitochondria-targeted self-assembled peptide-nanomaterials developed in recent years. The latest strategies and advances for constructing self-assembling peptides that target and assemble in mitochondria are highlighted, including the equipment of mitochondria-targeted ligands, the introduction of a stimuli-responsive mechanism to trigger the self-assembly *in situ*, and so on. Meanwhile, we briefly discuss the application of these nanomaterials in cancer therapy.

## Self-Assembling Peptide

**FIGURE 1:**
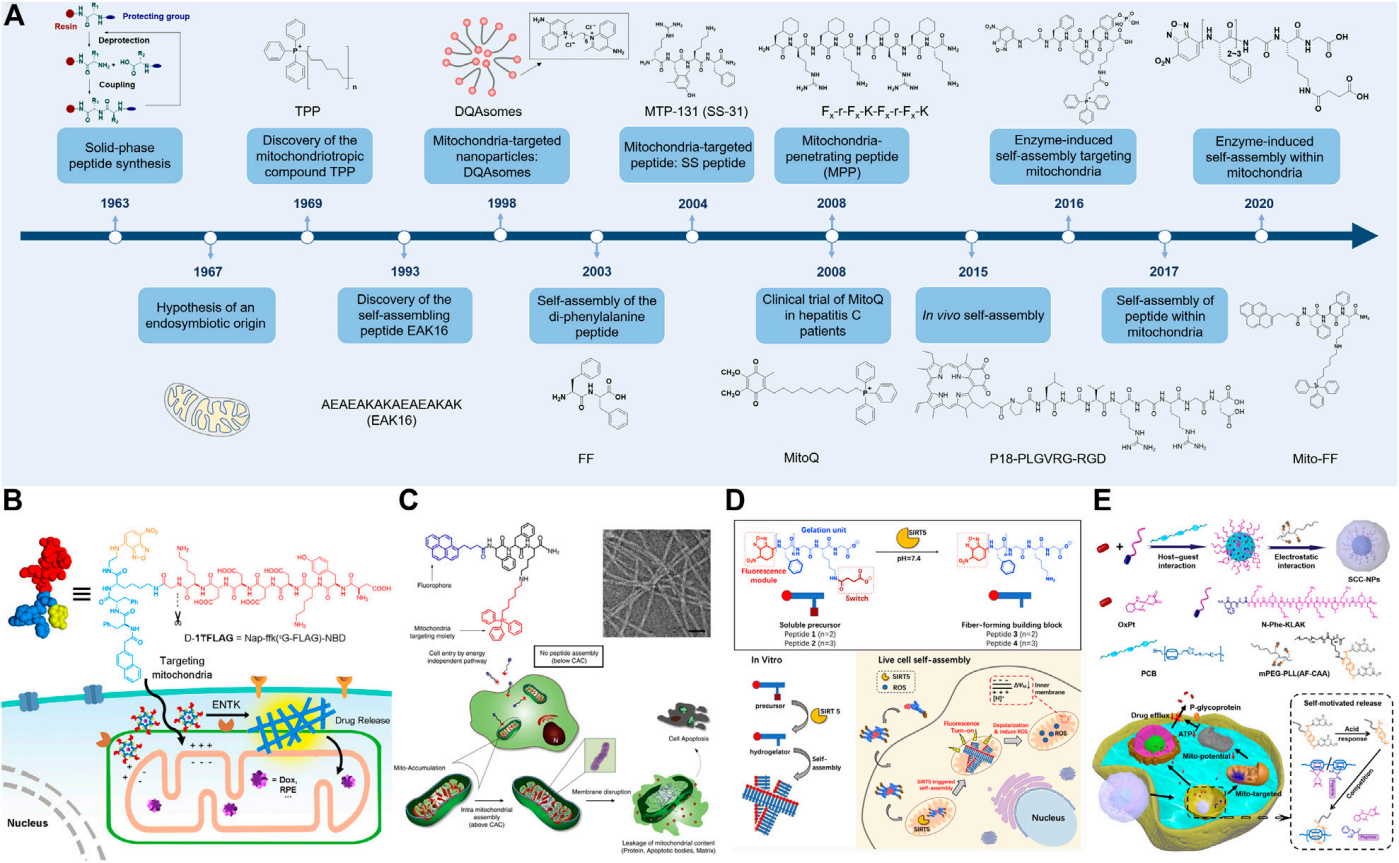
**(A)** A brief timeline of selected events for mitochondria-targeted self-assembly of peptide-nanomaterials. The references are shown in Supplementary Table S1. **(B)** *In situ* enzyme-instructed self-assembly of branched peptides around mitochondria. Reproduced with permission from ref (He et al., 2018). Copyright 2018 American Chemical Society. **(C)** *In situ* self-assembly of peptide amphiphiles in mitochondria due to the enhanced accumulation by targeting. Reproduced with permission under a Creative Commons CC BY License from ref (Jeena et al., 2017). Copyright 2017 Springer Nature. **(D)** *In situ* enzyme-instructed self-assembly of peptides in mitochondria. Reproduced with permission from ref (Yang L. et al., 2020). Copyright 2020 American Chemical Society. **(E)** Self-motivated release of the mitochondria-cytotoxic peptide to strengthen the chemotherapy toward drug-resistant cancer cells. This figure has been published in CCS Chemistry 2021; Self-Motivated Supramolecular Combination Chemotherapy for Overcoming Drug Resistance Based on Acid-Activated Competition of Host–Guest Interactions is available online at 10.31635/ccschem.021.202100964; https://www.chinesechemsoc.org/doi/10.31635/ccschem.021.202100964.

Peptides are usually defined as the biomacromolecules that contain less than 50 amino acids linked by peptide bonds, with the intrinsic characteristics of folding and bioactivities like recognition and response. Since the emergences of technologies for the peptide manufacture and screening, especially the solid-phase peptide synthesis proposed by Merrifield in 1963 (Merrifield, 1963) and the phage display described by Smith in 1985 (Smith, 1985), numerous peptide-based pharmaceuticals and functional materials have been developed (Figure 1A) (Hosoyama et al., 2019; Li et al., 2020; Lopez-Silva and Schneider, 2021; Muttenthaler et al., 2021). Particularly, self-assembling peptides have attracted increasing interest due to their improved stability and biological performance (Guyon et al., 2018; Levin et al., 2020), and have been applied in a wide range of fields including the tissue engineering (Gelain et al., 2020; Zhang et al., 2021), drug delivery (Moitra et al., 2014; Abbas et al., 2017; Yang J. et al., 2020; Kumar et al., 2020; Ji et al., 2021), catalysis (Rufo et al., 2014; Wang M. et al., 2020; Liu Q. et al., 2021; Chen et al., 2021), semi-conducting device (Tao et al., 2017), and energy materials (Hu et al., 2018; Lee et al., 2018; Nguyen et al., 2021). With the molecular basis to form secondary structures including the α-helix and β-sheet, the self-assembling peptides can assemble into well-defined nanostructures like nanofibrils driven by non-covalent interactions, such as the hydrophobic interaction, electrostatic interaction, π-π stacking, hydrogen bond, etc (Hendricks et al., 2017; Hu et al., 2020; Zheng et al., 2021). For instance, the di-phenylalanine peptide (FF), the most widely investigated self-assembling peptide, can assemble into either the nanofiber, nanotube, nanosphere, or nanoarray on the surface, by using properly mixed solvents and kinetic controls, or vapor deposition (Reches and Gazit, 2003; Wei et al., 2017; Zhao et al., 2019). The rapid development of self-assembling peptides can be traced back to 1990s (Zhang, 2020). The peptides in early researches are limited and mainly inspired from natural proteins especially amyloid proteins (Zhang, 2003), like FF and KLVFF. The developments of machine learning (Jumper et al., 2021) and one-bead one-compound (OBOC) combinatorial library (Lam et al., 1991) technologies make the screening of self-assembling peptides in a large-scale manner possible. For instance, Frederix et al. developed a computational simulation tool based on the aggregation propensity and amphiphilicity of peptides, to rapidly screen the self-assembling tripeptides in all 8,000 possible sequences and guided the discoveries of several unreported tripeptides that could form hydrogels (Frederix et al., 2015). In another work, Li et al. focused on the relationship between the chemical structures of peptides and their albitites to form hydrogels, applying machine learning to generate a hydrogel library with more than 2,000 self-assembling dipeptides (Li F. et al., 2019). Besides computational simulations (Moitra et al., 2017), Yang et al. recently capped a hydrophobicity-sensitive probe, the nitro-1,2,3-benzoxadiazole (NBD), to the N-terminus of peptides on beads in the OBOC library, achieving the rapid screening of self-assembling pentapeptides experimentally (Yang et al., 2021).

## Self-Assembled Peptide-Nanomaterials for Targeting Mitochondria

In recent years, targeted drug delivery systems have shown promising potentials in precision and personalized medicine, with reduced side effects (Yi et al., 2019; Mi et al., 2020; Manzari et al., 2021). In this regard, the integration with the biomarker-targeting, enzyme-response, and treatment makes the self-assembling peptides as candidates for functional nanomaterials and smart nanomedicines more than scaffolds in hydrogels. This endeavor has been promoted by the establishment of several important concepts including the peptide amphiphiles (Lowik and van Hest, 2004; Cui et al., 2010), the enzyme-instructed self-assembly (EISA) (Zhou and Xu, 2015; He et al., 2020c), and the *in vivo* self-assembly (Zhang et al., 2015; He P.-P. et al., 2020; Mamuti et al., 2021). Since mitochondria serve as a potential target for cancers (Guo et al., 2021; Liew et al., 2021), dozens of mitochondria-targeted self-assembled peptide-nanomaterials have been reported (Table 1), including the pre-assembled peptide-nanomaterials that locate mitochondria and *in situ* self-assembling peptides that assemble in mitochondria. In this section, we discuss the former.

**TABLE 1:** Recent progress of mitochondrial-targeted self-assembly of peptide-nanomaterials.

Materials	Peptide components^a^	Targeting mechanism	Assembling modules	Applications	References
Peptide amphiphile	Pyrene-FFK(TPP)	TPP ligand for targeting mitochondrial membrane	Pyrene-FF	Intra-mitochondrial assembly for cancer therapy *in vitro*	Jeena et al. (2017)
Peptide amphiphile	Pyrene-FFK(TPP) and pyrene-ffk (TPP)	TPP ligand for targeting mitochondrial membrane	Pyrene-FF and pyrene-ff	Treatment of colorectal tumor (HT-29) *in vivo*	(Jeena et al., 2019; Jeena et al., 2020b)
Peptide amphiphile	C_16_-MIASHLLAYFFTELN-KVLKQRAKKK	Targeting mitochondrial VDAC1 by the peptide (MIASHLLAYFFTELN) derived from hexokinase-II protein	C_16_ alkyl chain	Treatment of lung cancer (A549) cells *in vitro*	Liu et al. (2019)
Peptide amphiphile	DYKDDDDKGE(C_16_)_2_	Enterokinase-induced cleavage of peptide for drug release located at mitochondria	Lipid-like E (C_16_)_2_	Delivery of chloramphenicol to liver tumoral (HepG2) mitochondria *in vitro*	He et al. (2020b)
Peptide amphiphile	Cy5-KLVFF-TPP	TPP ligand for targeting mitochondrial membrane	KLVFF	Targeted NIR imaging and dysfunction of mitochondria in cervical and lung cancer (HeLa and A549) cells *in vitro*	Chandra Saha et al. (2020)
Peptide amphiphile	Pyrene-FFK(TPP)	TPP ligand for targeting mitochondrial membrane	Pyrene-FF	Treatment of sorafenib-resistant hepatocellular carcinoma (Huh7) cells *in vitro*	Hong et al. (2021)
Peptide amphiphile	Cy3-TPP/FF and Cy5-TPP/FF	TPP ligand for targeting mitochondrial membrane	FF	Mitochondria-targeted NIR imaging and early apoptosis of cancer cells *in vitro*	Saha et al. (2021)
Self-assembling peptide	NBD-FF_p_YK	TPP ligand for targeting mitochondrial membrane	NBD-FF	Treatment of osteosarcoma (Saos2) cells *in vitro*	Wang et al. (2016)
Self-assembling branched peptide	Nap-ffk (GDYKDDDDK)-NBD	Enterokinase-induced cleavage of peptide for self-assembly located at mitochondria	Nap-ffk(G)-NBD	Delivery of doxorubicin and red phycoerythrin to tumoral (HeLa) mitochondria *in vitro*	He et al. (2018)
Self-assembling peptide	NBD-FFFGK (succ)G and Fmoc-FFFGK (succ)G	Mitochondria-localized SIRT5 enzyme-induced desuccinylation of peptide for intra-mitochondrial self-assembly	NBD-FFF and Fmoc-FFF	Imaging of SIRT5 in living cells and improvement of the anticancer activities of dichloroacetate, cisplatin, and paclitaxel toward cervical cancer (HeLa) cells *in vitro*	Yang et al. (2020b)
Self-assembling peptide	Nap-ffk (GDYKDDDDK)y	Enterokinase-induced cleavage of peptide for self-assembly located at mitochondria	Nap-ffky	Delivery of histone protein H2B to tumoral (HeLa) mitochondria *in vitro*	He et al. (2020a)
Self-assembling peptide	PEG-thioketal-K(P18)-(KLAKLAK)_2_	ROS-triggered detachment of PEG to expose KLAK peptides for disrupting mitochondria	K(P18)-LVFF	Ultrasound-mediated treatment of orthotopic human pancreatic carcinoma (PANC-1) *in vivo*, and photoacoustic imaging-guided and NIR irradiation-mediated treatment of cervical tumor (HeLa) *in vivo*	(Cheng et al., 2020; Zhang et al., 2020)
Peptide/pDNA self-assembly	MLSLRQSIRFFK-(KH)_9_ and MLFNLRILLNNAAFRNGHNFMVRNFRCGQPLQ-(KH)_9_	Peptides derived from yeast Cytcox (MLSLRQSIRFFK) and human hepatic enzyme ornithine transcarbamylase (MLFNLRILLNNAAFRNGHNFMVRNFRCGQPLQ) for targeting mitochondria	Complexation of (KH)_9_ with pDNA *via* electrostatic interaction	Delivery of pDNA to cellular mitochondria *in vitro*	Chuah et al. (2016)
Polymer-peptide conjugate	CGGG-(KLAKLAK)_2_ and CGGG-(HLAHLAH)_2_	(KLAKLAK)_2_ or (HLAHLAH)_2_ peptides for disrupting mitochondrial membrane	Poly (β-thioester) polymeric backbone	Treatments of glioblastoma (U87) and cervical cancer (HeLa) cells *in vitro*, and murine melanoma (B16F10) *in vivo*	(Qiao et al., 2016b; Qiao et al., 2017a; Qiao et al., 2017b; Cheng et al., 2017; Cong et al., 2019)
Polymer-peptide conjugate	CGGG-(KLAKLAK)_2_ and CYGRKKRRQRRR	Cell-penetrating peptide CYGRKKRRQRRR for enhancing cellular uptake and KLAK peptide for disrupting mitochondrial membrane	PAMAM or poly (β-thioester) polymeric backbone	Treatments of glioblastoma (U87) cells *in vitro,* and breast tumor (SKBR-3) *in vivo* combined with photothermal therapy	(Liu et al., 2018a; Liu et al., 2018b)
Polymer-peptide conjugate	CGGG-(KLAKLAK)_2_ and CGGGKLVFF-thioketal-PEG	ROS triggered detachment of PEG to exposure the KLAK peptide for disrupting mitochondrial membrane	KLVFF conjugated poly (β-thioester)	Treatment of cervical tumor (HeLa) *in vivo*	Cheng et al. (2019)
Supramolecular polymer-peptide complex	Supramolecular complex assembled from FGG-(kalkalk)_2_ and PEG-cucurbit[7]uril copolymers through host-guest interaction	KLAK peptide for disrupting mitochondrial membrane	PEG-cucurbit[7]uril copolymers	Treatments of colorectal tumor (HCT116) *in vivo,* and drug-resistant HCT116 cancer cells *in vitro*	(Wang et al., 2020a; Wang et al., 2021a)

a Peptides are named by using the standard single-letter amino acid code. The capital letter refers to the L-type amino acid, whereas the lowercase letter means the D-type amino acid. The abbreviations used in the table include the triphenylphosphonium (TPP), voltage-dependent anion channel-1 (VDAC1), sirtuin 5 (SIRT5), nitro-1,2,3-benzoxadiazole (NBD), naphthalene (Nap), succinylated lysine [K (succ)] (KLAKLAK)_2_ (KLAK), near-infrared (NIR), reactive oxygen species (ROS), poly (ethylene glycol) (PEG), purpurin-18 (P18), plasmid deoxyribonucleic acid (pDNA), cytochrome c oxidase subunit IV (Cytcox), cyanine 3 and 5 (Cy3 and Cy5), and polyamidoamine (PAMAM).

A typical approach to construct the mitochondria-targeted self-assembling peptide is to equip a targeting motif to a self-assembling peptide. For instance, Standley et al. combined the α-helical (KLAKLAK)_2_ (KLAK) peptide, a cytotoxic peptide that breaks mitochondrial membranes (Ellerby et al., 1999), with a hydrophobic alkyl tail and a β-sheet forming peptide to afford a mitochondria-targeted peptide amphiphile (Standley et al., 2010). This peptide amphiphile could assemble into nanofibers, which were demonstrated to enter the breast cancer cells, then locate and disrupt the mitochondrial membranes. To increase the targeting efficacy, stimuli such as the endogenous enzyme, redox, and acidic environments, as well as the exogenous light and ultrasound, are employed to guide the targeted self-assemblies (Chen et al., 2018b; Jiang et al., 2021). For instance, Wang et al. reported an alkaline phosphatase (ALP)-instructed self-assembling peptide for targeting mitochondria (Wang et al., 2016). The peptide consisted of a mitochondria-targeting motif (TPP), a self-assembling backbone (FFYK), an enzyme-responsive phosphorylated tyrosine, and a fluorophore (NBD). Upon dephosphorylation by ALP overexpressed on cancer cell membranes, the peptide became more hydrophobic and self-assembled into nanofibers. After endocytosis and endosomal escape, the peptide-assemblies accumulated to mitochondria assisted by TPP, resulting in mitochondrial dysfunction and cell death. Notably, under the experimental concentration, the peptide did not form assemblies in the low ALP-expressed HS-5 (normal human bone marrow stromal) cells.

Despite the successes in cultured cells, the self-assembling peptides still face the intrinsic nature of instability in physiological environments, as well as several physiological barriers when applied in the body, such as the rapid clearance in blood, uncontrolled transportation to diseased tissues, insufficient cellular internalization and endosomal escape, etc (Sun et al., 2017). To improve the biological performances in the body, Wang and coworkers reported a series of polymer-peptide conjugates (PPCs) as *in vivo* self-assembled nanomedicines (Li LL. et al., 2019). For instance, Cong et al. reported a type of PPCs that targeted tumoral mitochondria with long blood circulation times (Cong et al., 2019). The PPCs consisted of a poly (β-thioester) backbone and KLAK peptides modified with pH-cleavable cis-aconitic anhydride (CAA) moieties and cell-penetrating peptides (TAT: CYGRKKRRQRRR) on the side chains. Upon systemic administration, the hydrophilic PPCs remained soluble as monomers for circulating long in the bloodstream and penetrating deeply into the solid tumors. Once arrived at the tumor tissue, the acidic tumor microenvironment trigged the cleaving of the hydrophilic CAA moieties on PPCs, resulting in the formation of 100 nm-sized nanoparticles decorated with KLAK and TAT peptides. These newly formed nanoparticles entered cancer cells facilitated by TAT peptides, then further located mitochondria and induced apoptosis associated with KLAK peptides.

## In Situ Self-Assembly of Peptide-Nanomaterials in Mitochondria

In the previous section, we discussed the assembled peptide-nanomaterials that locate mitochondria. Recently, the *in situ* self-assembly attracts much attention due to its spatiotemporal precision and activable bioeffects, emerging as a frontier in the biomedical field (Deng et al., 2021; Kwek et al., 2021; Liu et al., 2021a; Wang et al., 2021b). The specific enzymes and overexpressed ROS can be used to trigger the self-assembly around or on the surface of mitochondria *in situ*. As shown in Figure 1B, He et al. reported a mitochondrial enterokinase (ENTK)-instructed branched peptide for self-assembly around mitochondria (He et al., 2018). A hydrophilic ENTK-cleavable Flag-tag (DDDDK) was conjugated to the peptide scaffold, resulting in a micelle-like structure. After cell internalization, the micelles transformed into nanofibers mainly at mitochondria due to the enzymatic cleaving by ENTK. This construction of self-assembling peptides has successfully delivered the chloramphenicol and histone proteins (H2B) to mitochondria of cancer cells *in vitro* (He et al., 2020a; He et al., 2020b). Furthermore, to achieve mitochondria-targeting in the body, Cheng et al. constructed a type of ROS-trigged morphology-transformable PPCs driven by the responsive detachment of poly (ethylene glycol) (PEG) chains (Cheng et al., 2019). The PPCs consisted of a poly (vinyl alcohol) backbone with side chain-modifications of PEGylated KLVFF peptides linked by ROS-cleavable thioketals and KLAK peptides. After administration, the micelle-like PPCs transported in the bloodstream with the shield of PEG chains. Once closing to mitochondria, the over-generated ROS cleaved the thioketal linker to detach PEG chains, resulting in a transformation of micelles to nanofibers that exposed KLAK peptides to disrupt mitochondrial membranes.

To further achieve the precise self-assembly of peptides inside mitochondria, the targeted accumulation-induced assembly and the intramitochondrial protease-instructed assembly have shown promising potentials. Since enough concentration higher than the critical aggregation concentration (CAC) is the basis for molecular assemblies, making the self-assembling peptides with a spatial concentration above CAC in mitochondria selectively and a concentration below CAC in the cytoplasm is a feasible approach to achieve *in situ* self-assembly in mitochondria. For instance, Jeena et al. reported a system of *in situ* self-assembly (Mito-FF) that assembled inside mitochondria through targeted accumulation-induced assembly (Figure 1C) (Jeena et al., 2017). Mito-FF consisted of FFK peptide backbone with a fluorescent pyrene at the N-terminus and TPP at the lysine side chain, with a CAC of 60 µM. Using a culture medium containing 5 and 10 µM Mito-FF, the amphiphilic Mito-FF could efficiently and selectively gather inside mitochondria of cervical cancer (HeLa) cells with a concentration of 3 and 11 mM, respectively, thereby aggregating into nanofibers inside mitochondria *in situ*. Besides the targeted accumulation-induced aggregation, Yang et al. recently utilized the mitochondrial localization enzyme sirtuin 5 (SIRT5) to induce peptide self-assembly inside mitochondria *in situ* (Figure 1D) (Yang L. et al., 2020). The peptide had a backbone of FFFGKG, with a fluorescent probe NBD at the N-terminus and a succinylated lysine residue. Once entering into mitochondria, the peptide was desuccinylated by SIRT5 to become more hydrophobic, resulting in the formation of nanofibers inside mitochondria.

## Applications in Cancer Therapy

Although peptides and peptide-based vaccines and nanomaterials have been frequently reported for cancer therapy (Chiangjong et al., 2020; Malonis et al., 2020; Rong et al., 2020; Fan et al., 2021), the self-assembled peptide-nanomaterials especially those targeting mitochondria as anticancer reagents are still in the primary stage. Currently, approaches of these nanomaterials for cancer therapy mainly include the delivery of anticancer drugs, destructions of mitochondria by mitochondria-cytotoxic peptides or peptide-assemblies, and the combination with chemotherapy or photothermal therapy (PTT) (Supplementary Table S2). For instance, to deliver the chloramphenicol selectively to mitochondria, He et al. employed ENTK-instructed self-assembled peptide-nanoparticles as nanocarriers (He et al., 2020b). Once arrived at mitochondria, the hydrophilic Flag-tag on the peptide was cleaved by ENTK, resulting in the release of chloramphenicol to mitochondria. The chloramphenicol further interrupted the mitochondrial metabolism by inhibiting the synthesis of mitochondrial proteins, resulting in the release of cytochrome c for apoptosis. This self-assembled nanoparticle showed selective cytotoxicity to tumor cells, with lower half-maximal inhibitory concentration (IC_50_) values toward human hepatoma (HepG2) cells (54 µM) and HeLa cells (73 µM) than those for normal HS-5 cells (143 µM) and murine hepatocyte (AML12) cells (142 µM). The selectivity might be due to either the lack of ENTK enzymes in HS-5 cells or the less polarized mitochondria in AML12 cells. In addition, the peptide-assemblies in mitochondria also show strong anticancer activities. For instance, Jeena et al. showed that the co-assembly of Mito-FF and its mirror formulation Mito-ff led to nanofibers with a diameter of 100 nm, resulting in the enhanced antitumor efficacy in subcutaneous colorectal adenocarcinoma (HT-29)-bearing mouse model *via* intraperitoneal injections (Jeena et al., 2019).

To realize the mitochondria-cytotoxic peptides for improved cancer therapy, Qiao et al. developed a type of PPCs containing KLAK peptides prepared by Michael-type addition (Qiao et al., 2016a). This synthetic method allowed the facile conjugations of targeted and therapeutic peptides together with PEG chains to a polymer backbone, achieving both the improved biological stability and enhanced anticancer efficacy toward the subcutaneous glioblastoma (U87)-bearing mouse model. However, this covalent approach suffers from the long reaction time (e.g., 2 days) and the competitive reactions from the thiol and amine groups. To construct a fast and simple method for systemic delivery of peptide pharmaceuticals, Wang et al. based on the concept of non-covalent supramolecular chemotherapy (Chen et al., 2017; Chen et al., 2018a; Wu et al., 2021), proposed a strategy of supramolecular peptide therapeutics (Wang H. et al., 2020). Owning to the strong host-guest interaction between the cucurbit[7]uril (CB[7]) and N-terminal phenylalanine (N-Phe) residue in the peptide (binding constant ∼2 × 10^6^ M^−1^), the N-Phe-containing KLAK peptides were carried by the CB[7]-PEG copolymers in a simple (mixing in the aqueous solution) and fast (several minutes) manner with a high peptide encapsulation efficiency (>97%) under the peptide concentration of 0.5 mM. This strategy achieved prolonged blood circulation (25% remained at 1 h after intravenous injections compared with 13% for the peptide alone), enhanced tumor accumulation (2.8-fold enhancement), and increased anticancer efficacy (4-fold enhancement in the tumor inhibition rate) toward the subcutaneous colorectal tumor (HCT116)-bearing mouse model *via* intravenous injections, with minimal hematologic, hepatic, and nephric toxicities. In addition, to overcome the drug resistance of cancer cells, the same group further combined the oxaliplatin and KLAK peptide that disrupted ATP generations synergistically, in an acid-trigged on-demand drug release system (Figure 1E), achieving the improved anticancer activity toward oxaliplatin-resistant HCT116 cells, with IC_50_ decreased from 76.5 µM (oxaliplatin) to 31.2 µM (Wang H. et al., 2021). Besides chemotherapy, Zhang et al. utilized the photothermal effect irradiated by the near-infrared (NIR) light to promote the self-assembly of a purpurin-18-containing peptide, resulting in a four times increase in the self-assembly rate and a 2-fold enhancement in the tumor accumulation (Zhang et al., 2020).

## Conclusion and Outlook

The advances in nanotechnology and biotechnology have witnessed the great progress of the self-assembling peptides from simple scaffolds of hydrogels to smart nanomaterials for versatile biomedical applications, bridging the gap between simple synthetic molecules with sophisticated biological machinery in the body. Owning to the advantages including the excellent biocompatibility and the inclusivity for multiple biological and physicochemical activities, the self-assembling peptide achieves the self-assembled accuracy from levels of the tissue, through cells, to cellular organelles. However, several challenges still exist for the further development of mitochondria-targeted self-assembling peptides. The first is the precise self-assembly. Besides the membrane potential, enzyme, and ROS, other candidates such as the mitochondrial protein import machinery and nucleic acids (Zhao et al., 2021) are also promising targets. The second one is the characterization *in situ*. High-resolution, real-time, and *in situ* technologies are highly desirable to investigate the process and kinetics of the self-assembly in organelles. The third one is the rapid design and screening. The technology of machine learning may provide a high-throughput method to develop self-assembling peptides equipped with multiple bioactive and functional modules. Last but not least, biological safety should be highly concerned. Since the prolonged retention nature of the peptide nanofibers, careful studies in the degradation, metabolism, and long-term toxicology of the self-assembling peptides are important for their further clinical applications. Despite the challenges, we believe the self-assembled peptide-nanomaterials, especially the organelle-precise self-assembling peptides, will contribute to the new paradigms of biomedical technologies and products.
