# Supplementary material for: Mitochondria-Targeted Self-Assembly of Peptide-Based Nanomaterials
Source: Front Bioeng Biotechnol. 2021 Nov 26;9:782234. doi: 10.3389/fbioe.2021.782234 (PMC8664541; doi:10.3389/fbioe.2021.782234)
Supplement: Supplementary file 1 [file DataSheet1.docx]

Supplementary Material for

**Mitochondria-Targeted Self-Assembly of Peptide-Based Nanomaterials**

Zhen Luo^1,2†^, Yujuan Gao^2†^, Zhongyu Duan^1^*, Yu Yi^2,3^*, Hao Wang^2,3^*

^1^School of Chemical Engineering and Technology, Hebei University of Technology, Tianjin, 300130, China

^2^CAS Center for Excellence in Nanoscience, CAS Key Laboratory for Biomedical Effects of Nanomaterials and Nanosafety, National Center for Nanoscience and Technology (NCNST), No. 11 Beiyitiao, Zhongguancun, Beijing 100190, China

^3^University of Chinese Academy of Sciences, Beijing 100190, China

^†^These authors contributed equally to this work.

*Corresponding authors: Zhongyu Duan ([zyduan@hebut.edu.cn](mailto:zyduan@hebut.edu.cn)); Yu Yi ([yiyu@nanoctr.cn](mailto:yiyu@nanoctr.cn)); Hao Wang ([wanghao@nanoctr.cn](mailto:wanghao@nanoctr.cn))

**TABLE S1 |** References for the timeline of selected events for mitochondria-targeted self-assembly of peptide-nanomaterials.

| **Year** | **Event** | **Reference** |
| --- | --- | --- |
| 1963 | Establishment of the solid-phase peptide synthesis (SPPS) | (Merrifield, 1963) |
| 1967 | Hypothesis of an endosymbiotic origin | (Sagan, 1967) |
| 1969 | Discovery of the mitochondriotropic compound TPP | (Liberman et al., 1969) |
| 1993 | Discovery of the self-assembling peptide EAK16 | (Zhang et al., 1993) |
| 1998 | Fabrication of the mitochondria-targeted nanoparticles, DQAsomes | (Weissig et al., 1998) |
| 2003 | Report of the self-assembly of di-phenylalanine peptides | (Reches and Gazit, 2003) |
| 2004 | Discovery of the mitochondria-targeted peptides, Szeto-Schiller peptides | (Zhao et al., 2004) |
| 2008 | Discovery of the mitochondria-penetrating peptide (MPP) | (Horton et al., 2008) |
| 2008 | Clinical trial of MitoQ in hepatitis C patients | ClinicalTrials.gov (NCT00433108) |
| 2015 | Establishment of “*in vivo* self-assembly” | (Zhang et al., 2015) |
| 2016 | Enzyme-induced self-assembly of peptide-nanomaterials for targeting mitochondria | (Wang et al., 2016) |
| 2017 | In situ self-assembly of TPP-conjugated peptides inside mitochondria | (Jeena et al., 2017) |
| 2020 | Enzyme-induced self-assembly of peptides inside mitochondria in situ | (Yang et al., 2020) |

**TABLE S2 |** Approaches for mitochondria-targeted self-assembling peptide-nanomaterials toward cancer therapy.

| **Approaches for cancer therapy** | **Peptide components** | **Achievements** | **References** |
| --- | --- | --- | --- |
| Targeted delivery of anticancer drugs or biomacromolecules | Nap-ffk(GDYKDDDDK)-NBD | Delivery of doxorubicin and red phycoerythrin to tumoral (HeLa) mitochondria *in vitro*. | (He et al., 2018) |
|  | DYKDDDDKGE(C_16_)_2_ | Delivery of the chloramphenicol to liver tumoral (HepG2) mitochondria *in vitro*, resulting in the inhibition of mitochondrial protein synthesis and release of cytochrome c for cancer cell apoptosis. | (He et al., 2020) |
| Disruption of mitochondria induced by peptide self-assembly | NBD-FF_p_YK | Treatment of osteosarcoma (Saos2) cells *in vitro* without causing acquired drug resistance. The D-peptide-nanomaterials have a higher cytotoxicity than L-peptides due to the higher stability. The intracellular concentration of L-peptides increases at first 6 h and decreases with longer incubation times, whereas the intracellular concentration of D-peptides increases at first 6 h and remains almost constant for at least 24 h. | (Wang et al., 2016) |
|  | Pyrene-FFK(TPP) | Intra-mitochondrial assembly for cancer therapy *in vitro*, achieving the 600-1100-fold enhancement in the concentration within mitochondria than that in culture media. | (Jeena et al., 2017) |
|  | Pyrene-FFK(TPP) and pyrene-ffk(TPP) | Improved colorectal tumor (HT-29) therapy *in vivo*, through the heterochiral assembly of L-peptides and D-peptides. Both the D-peptides and the mixture of L- and D-peptides are more stable against the chymotrypsin than L-peptides. Upon treatment with 1 mg/mL chymotrypsin in the solution, D-peptides remain stable for at least 24 h. Meanwhile, only 49.5% and 42.8% L-peptides are retained for 12 and 24 h, respectively. Moreover, after an incubation time of 5 days, 74.6% of the mixture of L- and D-peptides with an equal molar ratio is retained, compared with 36.3% for L-peptides. | (Jeena et al., 2019;Jeena et al., 2020) |
|  | Cy5-KLVFF-TPP | Targeted NIR imaging and dysfunction of mitochondria in cervical and lung cancer (HeLa and A549) cells *in vitro*. | (Chandra Saha et al., 2020) |
|  | Pyrene-FFK(TPP) | Treatment of sorafenib-resistant hepatocellular carcinoma (Huh7) cells *in vitro*, through the up-regulation of mitochondrial ROS | (Hong et al., 2021) |
|  | Cy3-TPP/FF and Cy5-TPP/FF | Mitochondria-targeted NIR imaging and early apoptosis of cancer cells *in vitro*. | (Saha et al., 2021) |
|  | (_p_Y&Y)K-(KLAKLAK)_2_ | Improved cancer (HeLa) therapy *in vivo*, through the enzyme-induced self-assembly of peptides and phase separation of cell membranes that enhances the peptide drug internalization. | (Guo et al., 2021) |
| Disruption of mitochondria by mitochondria-cytotoxic peptides | Poly(β-thioester)s modified with CGGG-(KLAKLAK)_2_ and PEG on the side chains | Treatment of glioblastoma (U87) *in vitro*. The polymer-peptide conjugates (PPCs) form nanoparticles, displaying the remarkably improved cellular internalization and a 400-fold enhancement in the antitumor activity compared with the free (KLAKLAK)_2_ peptide. | (Qiao et al., 2016) |
|  | Poly(β-thioester)s modified with CGGG-(HLAHLAH)_2_ and detachable PEG by MMP-2 enzymes on the side chains | Treatment of glioblastoma (U87) *in vivo*. After systemic administration, the PEG chains protect the peptide-nanoparticles to circulate long in the bloodstream and accumulate in the tumor site effectively, meanwhile the (HLAHLAH)_2_ peptides remain as random coils with low cytotoxicity. Once arrived at tumor tissues, the PEG chains are cleaved by overexpressed MMP-2 enzymes to expose peptides, meanwhile the (HLAHLAH)_2_ peptides are activated by the acidic tumor microenvironment to be α-helical and cytotoxic, endowing the peptide-nanoparticles with enhanced antitumor activities. | (Qiao et al., 2017b) |
|  | Poly(amidoamine) (PAMAM) modified with CGGG-(KLAKLAK)_2_ | Treatment of glioblastoma (U87) *in vitro*, through the autophagy blockage by the PPCs due to the lysosome impairment as well as the mitochondrial damage synergistically. | (Qiao et al., 2017a) |
|  | Poly(β-thioester)s modified with CGGG-(KLAKLAK)_2_, cell-penetrating TAT peptides (CYGRKKRRQRRR), and detachable PEG by MMP-2 enzymes on the side chains | Treatment of glioblastoma (U87) cells *in vitro*. | (Liu et al., 2018b) |
|  | Poly(β-thioester)s modified with CGGG-(KLAKLAK)_2_ peptides containing pH-cleavable cis-aconitic anhydride moieties and cell-penetrating TAT peptides on the side chains | Treatment of murine melanoma (B16F10) *in vivo.* Upon systemic administration, the hydrophilic PPCs remain soluble as monomers for circulating long in the bloodstream and penetrating deeply into the solid tumors. Once arrived at the tumor tissue, the acidic tumor microenvironment triggers the cleaving of the hydrophilic CAA moieties on PPCs, resulting in the formation of 100 nm-sized nanoparticles decorated with KLAK and TAT peptides. These newly formed nanoparticles enter cancer cells facilitated by TAT peptides, and further locate mitochondria and induce apoptosis associated with KLAK peptides. | (Cong et al., 2019) |
|  | C_16_-MIASHLLAYFFTELN-KVLKQRAKKK | Treatment of lung cancer (A549) cells *in vitro*. | (Liu et al., 2019) |
|  | Poly(vinyl alcohol) (PVA) modified with CGGG-(KLAKLAK)_2_ and CGGGKLVFF-thioketal-PEG on the side chains | Treatment of cervical tumor (HeLa) *in vivo*. After systemic administration, the micelle-like PPCs transport in the bloodstream with the shield of PEG chains. Once closing to mitochondria, the over-generated ROS cleaves the thioketal linker to detach PEG chains, resulting in a transformation of micelles to nanofibers with KLAK peptides on the surface that rupture mitochondrial membranes effectively. | (Cheng et al., 2019) |
|  | Supramolecular self-assembly generated from FGG-(kalkalk)_2_ and PEG-cucurbit[7]uril (PEG-CB[7]) copolymers through host-guest interaction | Treatment of colorectal tumor (HCT116) *in vivo*. Owning to the strong host-guest interaction between the CB[7] and N-terminal phenylalanine (N-Phe) residue in the peptide (binding constant ~2 × 10^6^ M^-1^), the N-Phe-containing KLAK peptides are carried by the PEG-CB[7] copolymers in a simple (mixing in the aqueous solution) and fast (several minutes) manner with a high peptide encapsulation efficiency (> 97%) under the peptide concentration of 0.5 mM. This supramolecular self-assembly achieves the prolonged blood circulation (25% remained at 1 h after intravenous injections *vs.* 13% for the free peptide), enhanced tumor accumulation (2.8-fold enhancement), and increased anticancer efficacy (4-fold enhancement in the tumor inhibition rate) toward the subcutaneous HCT116 tumor-bearing mouse model via intravenous injections, with minimal hematologic, hepatic, and nephric toxicities. | (Wang et al., 2020) |
| Combination with chemotherapy | Poly(β-thioester)s modified with CGGG-(KLAKLAK)_2_ peptides ad PEG on the side chains | Treatment of cervical cancer (HeLa) cells *in vitro*. The loading of doxorubicin makes the PPCs more stable, meanwhile the PPCs enhance the antitumor activity of doxorubicin by breaking the tumoral mitochondria. | (Cheng et al., 2017) |
|  | NBD-FFFGK(succ)G and Fmoc-FFFGK(succ)G | Imaging of SIRT5 in living cells and improvement of the anticancer activities of dichloroacetate, cisplatin, and paclitaxel toward cervical cancer (HeLa) cells *in vitro*. | (Yang et al., 2020) |
|  | Supramolecular self-assembly generated from FGG-(kalkalk)_2_ and PEG-CB[7] copolymers through host-guest interaction | Treatment of drug-resistant HCT116 cancer cells *in vitro*. To overcome the drug resistance of cancer cells, the oxaliplatin is combined with the KLAK peptide that disrupts ATP generations synergistically, in an acid-trigged on-demand drug release system, achieving the improved anticancer activity toward oxaliplatin-resistant HCT116 cells, with a decreased IC_50_ (31.2 µM) compared with that of oxaliplatin (76.5 µM). Of note, this research proposes a concept of “self-motivated drug release” based on the acid-activate competition of host–guest interaction, resulting in the stable drug transportation and on-demand release (release rate > 93%). | (Wang et al., 2021) |
| Combination with photothermal therapy | PEG-thioketal-K(P18)-(KLAKLAK)_2_ | NIR irradiation-mediated treatment of cervical tumor (HeLa) *in vivo*. The photothermal effect irradiated by NIR light promotes the self-assembly of the peptide, resulting in the 4-fold increase in the self-assembly rate and 2-fold enhancement in the tumor accumulation. | (Zhang et al., 2020) |
|  | PEG-thioketal-K(P18)-(KLAKLAK)_2_ | Ultrasound-mediated treatment of orthotopic human pancreatic carcinoma (PANC-1) *in vivo*. | (Cheng et al., 2020) |
|  | Poly(β-thioester)s modified with CGGG-(KLAKLAK)_2_, TAT peptides, and the indocyanine green (ICG) on the side chains | Treatment of breast tumors (SKBR-3) *in vivo* combined with the photothermal therapy. In normal physiological environments, the PPCs as monodispersed molecules with a small size (< 10 nm) penetrate deeply in the tumor tissues. Upon NIR irradiation, the ICG converts light to heat, resulting in the in situ self-assembly of the thermoresponsive PPCs to form nanoparticles. These PPCs nanoparticles accumulate in tumors and enter cells effectively, achieving the improved cancer therapeutics. | (Liu et al., 2018a) |

**References**

Chandra Saha, P., Das, R.S., Chatterjee, T., Bhattacharyya, M., and Guha, S. (2020). Supramolecular beta-sheet forming peptide conjugated with near-infrared chromophore for selective targeting, imaging, and dysfunction of mitochondria. *Bioconjug. Chem.* 31, 1301-1306.

Cheng, D.-B., Yang, P.-P., Cong, Y., Liu, F.-H., Qiao, Z.-Y., and Wang, H. (2017). One-pot synthesis of pH-responsive hyperbranched polymer–peptide conjugates with enhanced stability and loading efficiency for combined cancer therapy. *Polym. Chem.* 8, 2462-2471.

Cheng, D.B., Zhang, X.H., Chen, Y., Chen, H., Qiao, Z.Y., and Wang, H. (2020). Ultrasound-activated cascade effect for synergistic orthotopic pancreatic cancer therapy. *iScience* 23, 101144.

Cheng, D.B., Zhang, X.H., Gao, Y.J., Ji, L., Hou, D., Wang, Z., Xu, W., Qiao, Z.Y., and Wang, H. (2019). Endogenous reactive oxygen species-triggered morphology transformation for enhanced cooperative interaction with mitochondria. *J. Am. Chem. Soc.* 141, 7235-7239.

Cong, Y., Ji, L., Gao, Y.J., Liu, F.H., Cheng, D.B., Hu, Z., Qiao, Z.Y., and Wang, H. (2019). Microenvironment-induced in situ self-assembly of polymer-peptide conjugates that attack solid tumors deeply. *Angew. Chem. Int. Ed.* 58, 4632-4637.

Guo, R.C., Zhang, X.H., Fan, P.S., Song, B.L., Li, Z.X., Duan, Z.Y., Qiao, Z.Y., and Wang, H. (2021). In vivo self-assembly induced cell membrane phase separation for improved peptide drug internalization. *Angew. Chem. Int. Ed.* doi: 10.1002/anie.202111839.

He, H., Lin, X., Guo, J., Wang, J., and Xu, B. (2020). Perimitochondrial enzymatic self-assembly for selective targeting the mitochondria of cancer cells. *ACS Nano* 14, 6947-6955.

He, H., Wang, J., Wang, H., Zhou, N., Yang, D., Green, D.R., and Xu, B. (2018). Enzymatic cleavage of branched peptides for targeting mitochondria. *J. Am. Chem. Soc.* 140, 1215-1218.

Hong, T.H., Jeena, M.T., Kim, O.H., Kim, K.H., Choi, H.J., Lee, K.H., Hong, H.E., Ryu, J.H., and Kim, S.J. (2021). Application of self-assembly peptides targeting the mitochondria as a novel treatment for sorafenib-resistant hepatocellular carcinoma cells. *Sci. Rep.* 11, 874.

Horton, K.L., Stewart, K.M., Fonseca, S.B., Guo, Q., and Kelley, S.O. (2008). Mitochondria-penetrating peptides. *Chem. Biol.* 15, 375-382.

Jeena, M.T., Jeong, K., Go, E.M., Cho, Y., Lee, S., Jin, S., Hwang, S.W., Jang, J.H., Kang, C.S., Bang, W.Y., Lee, E., Kwak, S.K., Kim, S., and Ryu, J.H. (2019). Heterochiral assembly of amphiphilic peptides inside the mitochondria for supramolecular cancer therapeutics. *ACS Nano* 13, 11022-11033.

Jeena, M.T., Lee, S., Barui, A.K., Jin, S., Cho, Y., Hwang, S.W., Kim, S., and Ryu, J.H. (2020). Intra-mitochondrial self-assembly to overcome the intracellular enzymatic degradation of L-peptides. *Chem. Commun.* 56, 6265-6268.

Jeena, M.T., Palanikumar, L., Go, E.M., Kim, I., Kang, M.G., Lee, S., Park, S., Choi, H., Kim, C., Jin, S.M., Bae, S.C., Rhee, H.W., Lee, E., Kwak, S.K., and Ryu, J.H. (2017). Mitochondria localization induced self-assembly of peptide amphiphiles for cellular dysfunction. *Nat. Commun.* 8, 26.

Liberman, E.A., Topaly, V.P., Tsofina, L.M., Jasaitis, A.A., and Skulachev, V.P. (1969). Mechanism of coupling of oxidative phosphorylation and the membrane potential of mitochondria. *Nature* 222, 1076-1078.

Liu, D., Angelova, A., Liu, J., Garamus, V.M., Angelov, B., Zhang, X., Li, Y., Feger, G., Li, N., and Zou, A. (2019). Self-assembly of mitochondria-specific peptide amphiphiles amplifying lung cancer cell death through targeting the VADC1-hexokinase-II complex. *J. Mater. Chem. B* 7, 4706-4716.

Liu, F.H., Cong, Y., Qi, G.B., Ji, L., Qiao, Z.Y., and Wang, H. (2018a). Near-infrared laser-driven in situ self-assembly as a general strategy for deep tumor therapy. *Nano Lett.* 18, 6577-6584.

Liu, F.H., Hou, C.Y., Zhang, D., Zhao, W.J., Cong, Y., Duan, Z.Y., Qiao, Z.Y., and Wang, H. (2018b). Enzyme-sensitive cytotoxic peptide-dendrimer conjugates enhance cell apoptosis and deep tumor penetration. *Biomater. Sci.* 6, 604-613.

Merrifield, R.B. (1963). Solid phase peptide synthesis. I. The synthesis of a tetrapeptide. *J. Am. Chem. Soc.* 85, 2149-2154.

Qiao, Z.Y., Lai, W.J., Lin, Y.X., Li, D., Nan, X.H., Wang, Y., Wang, H., and Fang, Q.J. (2017a). Polymer-KLAK peptide conjugates induce cancer cell death through synergistic effects of mitochondria damage and autophagy blockage. *Bioconjug. Chem.* 28, 1709-1721.

Qiao, Z.Y., Zhao, W.J., Cong, Y., Zhang, D., Hu, Z., Duan, Z.Y., and Wang, H. (2016). Self-assembled ROS-sensitive polymer-peptide therapeutics incorporating built-in reporters for evaluation of treatment efficacy. *Biomacromolecules* 17, 1643-1652.

Qiao, Z.Y., Zhao, W.J., Gao, Y.J., Cong, Y., Zhao, L., Hu, Z., and Wang, H. (2017b). Reconfigurable peptide nanotherapeutics at tumor microenvironmental pH. *ACS Appl. Mater. Interfaces* 9, 30426-30436.

Reches, M., and Gazit, E. (2003). Casting metal nanowires within discrete self-assembled peptide nanotubes. *Science* 300, 625-627.

Sagan, L. (1967). On the origin of mitosing cells. *J. Theor. Biol.* 14, 225-274.

Saha, P.C., Bera, T., Chatterjee, T., Samanta, J., Sengupta, A., Bhattacharyya, M., and Guha, S. (2021). Supramolecular dipeptide-based near-infrared fluorescent nanotubes for cellular mitochondria targeted imaging and early apoptosis. *Bioconjug. Chem.* 32, 833-841.

Wang, H., Feng, Z., Wang, Y., Zhou, R., Yang, Z., and Xu, B. (2016). Integrating enzymatic self-assembly and mitochondria targeting for selectively killing cancer cells without acquired drug resistance. *J. Am. Chem. Soc.* 138, 16046-16055.

Wang, H., Wu, H., Yi, Y., Xue, K.-F., Xu, J.-F., Wang, H., Zhao, Y., and Zhang, X. (2021). Self-motivated supramolecular combination chemotherapy for overcoming drug resistance based on acid-activated competition of host–guest interactions. *CCS Chem.* 3, 1413-1425.

Wang, H., Yan, Y.-Q., Yi, Y., Wei, Z.-Y., Chen, H., Xu, J.-F., Wang, H., Zhao, Y., and Zhang, X. (2020). Supramolecular peptide therapeutics: Host–guest interaction-assisted systemic delivery of anticancer peptides. *CCS Chem.* 2, 739-748.

Weissig, V., Lasch, J., Erdos, G., Meyer, H.W., Rowe, T.C., and Hughes, J. (1998). DQAsomes: A novel potential drug and gene delivery system made from dequalinium. *Pharm. Res.* 15, 334-337.

Yang, L., Peltier, R., Zhang, M., Song, D., Huang, H., Chen, G., Chen, Y., Zhou, F., Hao, Q., Bian, L., He, M.L., Wang, Z., Hu, Y., and Sun, H. (2020). Desuccinylation-triggered peptide self-assembly: Live cell imaging of SIRT5 activity and mitochondrial activity modulation. *J. Am. Chem. Soc.* 142, 18150-18159.

Zhang, D., Qi, G.B., Zhao, Y.X., Qiao, S.L., Yang, C., and Wang, H. (2015). In situ formation of nanofibers from purpurin18-peptide conjugates and the assembly induced retention effect in tumor sites. *Adv. Mater.* 27, 6125-6130.

Zhang, S., Holmes, T., Lockshin, C., and Rich, A. (1993). Spontaneous assembly of a self-complementary oligopeptide to form a stable macroscopic membrane. *Proc. Natl. Acad. Sci. U. S. A.* 90, 3334-3338.

Zhang, X.H., Cheng, D.B., Ji, L., An, H.W., Wang, D., Yang, Z.X., Chen, H., Qiao, Z.Y., and Wang, H. (2020). Photothermal-promoted morphology transformation in vivo monitored by photoacoustic imaging. *Nano Lett.* 20, 1286-1295.

Zhao, K., Zhao, G.M., Wu, D., Soong, Y., Birk, A.V., Schiller, P.W., and Szeto, H.H. (2004). Cell-permeable peptide antioxidants targeted to inner mitochondrial membrane inhibit mitochondrial swelling, oxidative cell death, and reperfusion injury. *J. Biol. Chem.* 279, 34682-34690.
